# Public engagement: Faculty lived experiences and perspectives underscore barriers and a changing culture in academia

**DOI:** 10.1371/journal.pone.0269949

**Published:** 2022-06-15

**Authors:** Mikhaila N. Calice, Becca Beets, Luye Bao, Dietram A. Scheufele, Isabelle Freiling, Dominique Brossard, Noah Weeth Feinstein, Laura Heisler, Travis Tangen, Jo Handelsman

**Affiliations:** 1 Department of Life Sciences Communication, University of Wisconsin-Madison, Madison, Wisconsin, United States of America; 2 Morgridge Institute for Research, Madison, Wisconsin, United States of America; 3 Department of Communication, University of Utah, Salt Lake City, Utah; 4 Department of Curriculum and Instruction, University of Wisconsin-Madison, Madison, Wisconsin, United States of America; 5 Robert E. and Jean F. Holtz Center for Science and Technology Studies, University of Wisconsin-Madison, Madison, Wisconsin, United States of America; 6 Wisconsin Alumni Research Foundation, Madison, Wisconsin, United States of America; 7 Wisconsin Institute for Discovery, University of Wisconsin-Madison, Madison, Wisconsin, United States of America; Ghent University: Universiteit Gent, BELGIUM

## Abstract

The idea of faculty engaging in meaningful dialogue with different publics instead of simply communicating their research to interested audiences has gradually morphed from a novel concept to a mainstay within most parts of the academy. Given the wide variety of public engagement modalities, it may be unsurprising that we still lack a comprehensive and granular understanding of factors that influence faculty willingness to engage with public audiences. Those nuances are not always captured by quantitative surveys that rely on pre-determined categories to assess scholars’ willingness to engage. While closed-ended categories are useful to examine which factors influence the willingness to engage more than others, it is unlikely that pre-determined categories comprehensively represent the range of factors that undermine or encourage engagement, including perceptual influences, institutional barriers, and scholars’ lived experiences. To gain insight into these individual perspectives and lived experiences, we conducted focus group discussions with faculty members at a large midwestern land-grant university in the United States. Our findings provide context to previous studies of public engagement and suggest four themes for future research. These themes affirm the persistence of institutional barriers to engaging with the public, particularly the expectations in the promotion process for tenure-track faculty. However, we also find a perception that junior faculty and graduate students are challenging the status quo by introducing a new wave of attention to public engagement. This finding suggests a “trickle-up” effect through junior faculty and graduate students expecting institutional support for public engagement. Our findings highlight the need to consider how both top-down factors such as institutional expectations and bottom-up factors such as graduate student interest shape faculty members’ decisions to participate in public engagement activities.

## Introduction

Interest in public engagement by scientists, and academic faculty more generally, is expanding across a range of disciplines as the scientific community becomes increasingly vocal about the necessity of public engagement with science [1, 2]. Engagement is especially important when emerging issues such as COVID-19 [e.g., 3], human genome editing [e.g., 4], and artificial intelligence [e.g., 5] raise questions in public debate that cannot be answered solely by more scientific research.

Responding to this urgent need for broad social debates, scholars have studied several aspects of public engagement, including different categories and levels of engagement, goals and outcomes, motivations and barriers, and the impact of training on willingness to engage [4, 6–9]. Despite this extensive body of work, there is little indication that academic faculty are devoting more time or resources to public engagement [10]. This suggests that research on public engagement does not fully capture the rich context of intersecting incentives and barriers that scientists face when trying to engage different sectors of the public around emerging science. As the need for more and better public engagement intensifies, it is important to understand the barriers that scientists face when deciding whether to engage with the public.

As disciplinary experts, university faculty are increasingly expected to engage with the public. Their perspectives about whether and why they choose to engage with the public are valuable for understanding how engagement can become more widespread. While some public engagement research explores faculty perspectives about specific aspects of engagement [e.g., 8], their views are rarely contextualized as explanations for why faculty choose to (not) engage. For example, previous work shows that there is a potential for institutional factors to influence faculty’s public engagement [e.g., 11, 12], but it remains unclear what these specific factors are and how they influence public engagement. Survey research in this space tends to focus on scientists’ attitudes toward public engagement under pre-determined categories [e.g., 13], but it does not represent faculty perspectives comprehensively with contextual factors such as the influence of institutional culture on participation in engagement activities [e.g., 14–16]. Examining the contextual factors provides a clearer picture of the reality of faculty experience.

In this study, we explore faculty perspectives and experiences with public engagement through qualitative focus group discussions with faculty members at a midwestern U.S. land-grant university. Land-grant universities are especially relevant cases for public engagement research because of their engagement-focused missions [17]. Our approach provides insight into how faculty view public engagement *at their institution* and into perspectives that are not captured by current public engagement research. This exploratory analysis builds upon previous research to consider a new framework of effective public engagement that is centered on goals and outcomes.

## Understanding the factors influencing willingness to do public engagement

Before diving into the factors that influence participation in engagement activities, we will briefly touch on what we mean by public engagement. Public engagement scholarship highlights the evolution of engagement from knowledge-deficit approaches to more complex definitions that consider the context of socially constructed knowledge [for a thorough review, see 18]. Seminal work on this in the field of science communication defines models of public engagement with stakeholders [e.g., 19] or identifies specific types of activities that fit within broader models [e.g., see 20]. Distinctions in the forms of engagement are still discussed, with specific attention to broad categories of engagement, as well as specifying goals related to why scientists engage with the public on certain issues [4].

There are various factors that influence why faculty might choose to partake in these various iterations of public engagement. These include the intrinsic, or individual, motivations and barriers, as well as extrinsic factors, such as incentives and expectations associated with university tenure or evaluation [e.g., 11]. Academic age (i.e., years since PhD completion, which can be roughly determined by tenure status) and department can also influence participation in engagement activities [e.g., 21, 22]. Furthermore, recent research identifies specific goals that can explain motivations for public engagement [e.g., 4, 23]. Understanding how faculty weigh these various factors provides greater context to the current state of public engagement research.

### Intrinsic factors motivate faculty participation in public engagement at a personal level

Intrinsic motivations for public engagement include individual factors that influence willingness to engage, such as personal enjoyment or a sense of responsibility [24]. Similarly, individuals who are motivated by the public good are more likely to talk with reporters and non-scientists [25]. The notion of engagement as a public good aligns with the belief that faculty should be held accountable to the public because their research is taxpayer funded [26] or because science has a social contract with society [27]. In a 2018 survey based on representative samples across 46 land-grant universities in the U.S., a majority of faculty were motivated to engage with the public by intrinsic rewards such as a sense of duty or a personal commitment (85%) and personal satisfaction or enjoyment (83%). Furthermore, most faculty (80%) disagreed that public engagement is not the job of scientists [10]. A 2013 study of faculty found that 86% of respondents similarly disagreed that political or public engagement is not the role of academics [28]. We build on this past research to explore whether these individual factors, including motivations and barriers, influence faculty participation in public engagement activities:

RQ1: How do faculty perceive the role of individual factors in their participation in public engagement activities?

### Perceptions that extrinsic factors create barriers and lack incentives for engagement

Extrinsic factors deal with the potential tensions between public engagement and faculty members’ research success. Some activities, like posting on Twitter, may increase research visibility [29], potentially leading to greater access to funding opportunities. On the other hand, some faculty perceive public engagement as an opportunity cost that diverts their time and money away from the research expected from them [21], although public engagement was not associated with reduced time and money when controlling for past experience [30]. Faculty also expressed concern about the risk of damaging their research reputation, for example, from misrepresentation of their research by the media or through criticism from their colleagues [31].

In comparison to intrinsic factors, research shows that faculty are less motivated by extrinsic factors such as demonstrating university research relevance (66%), meeting service requirements by their university appointment (45%), or obtaining funding (40%) [10]. Similarly, some faculty are concerned about potential drawbacks to their reputation and career, including making them a target (38% agree; 33% disagree), not helping their career (27% agree; 44% disagree), and making them less involved in their research (60% disagree) [10]. Other research found that four in ten faculty believed public engagement is time-consuming and distracting, and about a third considered it to be dangerous due to misquotes [28].

A subset of external factors specific to academic institutions, which we refer to as *institutional factors*, become more important in faculty decisions to engage when faculty are constrained by time and scholarly duties required by tenure and promotion [21]. Notably, institutional barriers are relatively prominent across multiple datasets, with a majority of scientists reporting a lack of institutional incentives to engage [54%; 10] or that public engagement is not valued by tenure committees [56%; 28]. Institutional factors require further attention since tensions resulting from institutional culture and structure still exist. Therefore, we ask:

RQ2: How do faculty perceive the role of institutional factors in their participation in public engagement activities?

### The role of tenure status and departmental culture in shaping willingness to engage

Higher education institutions are at the center of scholarly development and practice. As a result, *institutional culture* can influence how faculty approach their scholarship. In this paper, we explore the influences of institutional culture on public engagement. We define institutional culture as the external (i.e., not personal) factors at a university that impact faculty perception of the value placed on public engagement at their university. In this sense, institutional culture includes how faculty internalize their own career goals and how they understand university expectations for teaching, research, and service in tenure and promotion.

Our conceptualization of institutional culture stems from previous research that shows how the culture of a university can influence faculty capacity to engage with the public. A study exploring how faculty are “socialized” with regard to public service at a land-grant university defines institutional culture as one means of socialization, associating it with how faculty perceive the value of public service in the tenure and promotion process [14]. The authors of that study further discuss institutional culture as being influenced by the university’s land-grant status and prominence as a research institution. Additionally, public engagement activities have been examined by their “organizational status” and “institutional relevance” [12]. More recently, qualitative interviews found a “positive engagement culture” exists where scientists have “positive attitudes, norms, and self-efficacy beliefs about participating in engagement activities” [32, p. 228]. In this study, we explore the role of tenure status and department-level culture as factors related to a broader institutional culture.

The departmental culture surrounding public engagement encompasses various actors and resources such as department leaders, colleagues, rewards, and availabilitiy of engagement opportunities. Department leaders and colleagues form normative beliefs about how engagement activities are valued [33]. Support from the head of a department is particularly important for junior scientists to participate in more public engagement activities [13]. One study found that colleagues’ frequent participation in engagement activities is associated with scientists’ higher willingness to engage with the public [30], although other research did not find similar effects [22]. Other factors such as generating revenue or receiving rewards and recognition (both departmental and individual) encourage faculty participation in engagement activities [13].

How motivational intrinsic rewards are considered to be can differ by academic age, given potential differences in levels of experience. For example, senior scientists are more motivated by a sense of duty, whereas younger scientists are more motivated by personal satisfaction and enjoyment [24]. However, previous research also indicates that pre-tenure or junior faculty are more likely to be constrained by tenure and promotion guidelines and have less autonomy to devote time to engagement activities, especially at universities that have no institutional incentives or rewards for engagement [e.g., 11, 31, 33]. Additionally, for senior faculty, tenure status offers more flexibility to engage with the public and a higher tolerance for concerns about potential consequences that could result from that engagement [28].

Institutional factors also interact. For example, in universities where tenure guidelines emphasize engagement and outreach, the effect of training on participation in certain engagement activities among senior scientists is stronger than in universities with less emphasis on engagement [34]. Compared with senior faculty, however, junior faculty perceive significantly greater importance in pursuing public engagement activities [9]. Additionally, junior faculty are more familiar and comfortable with using new technology, like social media, to engage with a broader public more efficiently [6, 35]. Online engagement can have positive effects on metrics of academic impact, such as the h-index. For instance, a recent study showed that mentions of a scientist’s research on Twitter were associated with a higher h-index [29]. This suggests that newer avenues for engagement (i.e., social media) are beginning to influence metrics that already hold value in academic career promotion. Furthermore, the differences in interest in engagement may depend, in part, on academic age (tenure status) and departmental culture. How these factors relate to willingness to engage requires further in-depth investigation, leading us to ask:

RQ3: Do faculty perceptions of participating in public engagement vary based on their tenure status and departmental culture?

### Goals of public engagement

The extensive foundation of public engagement research continues to be further developed, especially for controversial science issues. A recent framework of effective public engagement [4] categorizes seven goals of public engagement activities. Although this framework was developed using the example of the genome editing technology, CRISPR, the goals are broad and may drive faculty perceptions of public engagement generally.

The first goal in this framework is that researchers might try to *avoid potential controversy* when promoting and garnering public acceptance for new technologies [36]. Although this goal may be well-intentioned, minimizing the communication of risks and benefits to the public could hinder deliberation and ultimately poses ethical problems. Second, the goal of *educating the public* assumes that the public only needs to know what scientists know in order to agree with them. There is evidence that increasing the public’s knowledge is not enough to change their minds, because people often rely on values, attitudes, and other biases to make judgments [37, 38]. Furthermore, those efforts can backfire [39]. Surveys of scientists, however, show that defending science and informing the public are of high priority, suggesting that scientists continue to operate within the knowledge deficit model [8]. Third, researchers might want to engage to *build democratic capacity through deliberation*. Successful deliberation assumes a willingness of individuals to consider attitude-inconsistent information [40], which can make the publics more tolerant and knowledgeable. This, in turn, can lead to valuable input that can influence, for example, the policymaking process. Of course, this “is based on the implicit assumption that all relevant voices in society are being heard in public debate” [4].

The fourth goal of the framework is that scientists might want to *widen the representation of voices* in public debate by including affected perspectives and voices that would not have otherwise been heard. The fifth goal presented is to *solicit input on value debates triggered by science*. Value debates that ideally include all affected stakeholders might center around ethical, legal, or social implications of research and can inform policymaking. Sixth, researchers might engage with the public because of science’s social contract with society (through investments in science; [27]), thus aiming at *enabling responsible innovation*. This involves continuous input from affected publics into the research process, which ideally prevents spending too much on specific developments or other unnecessary research. The last goal is to *shape policy*. This goal can take many forms, such as providing testimony or advising political representatives. Examples such as the Danish Consensus Conferences or formal reports by the National Academies in the United States are designed to bring in expert voices to advise policymakers.

The framework described above classifies the goals of public engagement based on previous research and on implicit or explicit assumptions underlying public engagement efforts. However, we do not know how well faculty perceptions map onto these goals and if there are other important goals in public engagement that faculty might pursue. We therefore ask:

RQ4: Are the goals of public engagement, as outlined in scholarship, reflected in faculty perspectives of and experiences with public engagement?

RQ5: Do other faculty goals emerge that are not covered by previous research?

## Methods

To answer our research questions, we conducted four 90-minute virtual focus groups that included 23 tenure-track faculty from a midwestern U.S. land-grant university from May to June 2020. The focus groups were designed to explore perspectives and experiences of faculty with public engagement that have not been widely addressed in previous research as well as to capture potential differences among faculty based on both academic age and the engagement level of their departments. The interview questions were developed based on where we saw opportunities for further research in the public engagement literature discussed in the previous section. After completion of the focus groups, we conducted a qualitative content analysis of the interview transcripts to reveal new themes, as well as to identify areas in which faculty perceptions of public engagement align with the current state of the research.

### Focus group design and recruitment process

The focus groups were designed to represent faculty of varying academic age (pre-tenure vs. tenured faculty) across departments organized by engagement level (high vs. low engaged departments). This resulted in four focus groups: pre-tenure high engagement, pre-tenure low engagement, tenured high engagement, and tenured low engagement. This approach required that we first categorized departments into high engagement and low engagement groups. Based on a 2018 survey collected at the same university, we aggregated the degree of faculty participation in engagement activities to the departmental level. We designed our focus groups this way in order to explore potential differences across the four interview groups. Excluding departments with fewer than four responses, we selected the top and bottom five to eight departments within each division based on the frequency indicator. To recruit participants from a wide range of departments and divisions, we then randomly selected two to three pre-tenure and tenured faculty in each department from their websites and collected their contact information for our recruitment sample.

In our initial sampling pool, we excluded faculty that held extension appointments. At land-grant institutions, extension appointment refers to formal appointments that require some form of engagement with communities throughout the state [41]. Our faculty perceptions of public engagement might have otherwise been skewed by the inclusion of extension faculty since they may experience different motivations and barriers than non-extension faculty. In fact, previous research shows that having an extension appointment is positively associated with more frequent participation in public engagement activities [42]. Excluding extension faculty allowed us to focus on perspectives from faculty who are not formally expected to engage with the public based on their appointment, but rather must choose to engage with the public on their own accord. Thus, we specifically included only non-extension, tenure-track faculty.

Our final sample included 188 faculty. Between April and May 2020, a representative at the University Survey Center recruited potential participants by inviting faculty from the sample via email until the target number of six participants for each focus group was achieved. The final sample included 23 participants representing all four university divisions (seven from Arts & Humanities, six from Biological Sciences, six from Physical Sciences, and four from Social Sciences) from 20 different departments. There were 11 pre-tenure and 12 tenured faculty. No incentives were provided and we made clear that participation was completely voluntary.

We acknowledge that there was potential for sampling error given that some of the selected faculty from low-engagement departments may be active participants in public engagement activities and vice versa. There is also a possibility of selection bias in that the faculty that volunteered to participate did so because of their existing interests in and experience with public engagement. The potential selection bias concerns will not substantially influence our analyses since individual faculty experiences provide legitimate insight into how departmental culture and tenure status might impact participation in public engagement.

### Interviews

The focus group interviews were conducted between May and June 2020, in a virtual format due to the restrictions imposed by the COVID-19 pandemic. Each of the four semi-structured interviews lasted about 90 minutes and were moderated by a senior qualitative research expert at the University Survey Center. A graduate-student member of the research team was present during each of the focus groups and introduced to the participants as a student observer. The moderator followed written guidelines and a set of questions during the discussions. The question guide was iteratively developed by all members of the research team and in collaboration with the professional moderator from the University Survey Center. While the moderator collaborated on the questions, with the aim of guiding the discussion, the moderator was not included in the other steps of the study, such as study design or analysis.

The questions used to guide the focus group discussions were developed based on previous public engagement research examining various individual and institutional factors that influence willingness to engage [11]. The questions covered three main themes of interest based on previous literature: broad perceptions of what engagement means to the participants, factors that impact their willingness to engage in various activities, and audience preferences (for details of the question guide, see S1 Appendix). The interviews were conducted virtually via Zoom, audio recorded, and professionally transcribed by the Survey Center. We included five to six people in each focus group in order to balance our interest in collecting diverse views while also providing adequate opportunities for each participant to express their opinions. During the transcription process, all identifying information about participants was removed.

Given that the design of the focus groups was informed by existing theory with a goal of discovering how those concepts mapped to the experiences of faculty at a particular institution, we were not trying to achieve data saturation, as would be the goal if we used grounded theory or phenomological approaches [e.g., 43, 44]. Because the focus groups were conducted at a single university, our sample is also inherently constrained and does not represent the broader population of tenure track faculty in the United States. Where the focus groups did introduce new, emergent engagement concepts, these should ultimately be viewed in the context of how they align with or add to concepts from previous engagement research and be framed within the context of this particular university.

### Analysis

We conducted qualitative data analysis with the software MAXQDA to uncover key themes from the focus group transcripts, using a combination of deductive and inductive approaches in our process. Deductively, we developed principal and sub-themes from previous literature and coded these as broad categories [45]. Inductively, we further coded any novel themes that appeared during the focus group discussions. When a new theme emerged, we recoded all transcripts to include instances in which the theme appeared. Throughout this coding process, several themes were identified, each with emerging sub-themes. Major themes related to public engagement included definitions and categorizations, motivations, barriers, institutional factors, target audiences, mentorship, and graduate student influence. The research team conducting the coding included three graduate student researchers. The researchers coded based on assigned themes across all four transcripts. Coding was then systematically reviewed by the other researchers. When new themes emerged, researchers noted these and met to discuss how they would be categorized before repeating the coding and systematic review process for the new themes. In total, we coded 547 perspectives on engagement across 19 coding categories and merged into themes that we report in the following sections.

### Public engagement among scientists: Perspectives and lived experiences

The focus groups provided additional context to previous research from individual faculty experiences and highlighted less obvious perspectives that require further investigation. In our findings, we first report on how these focus group discussions build upon existing research. We address research questions one, two, and three, which touch on factors that impact willingness to engage, including motivations and barriers to participating in engagement, such as tenure status and departmental culture. We then report findings specific to research question four, which considers whether participants were motivated by any of the goals identified in recent research, or by new goals not yet identified. Lastly, we detail four themes that emerged that represent relevant perspectives for additional future public engagement research. These four themes include (1) the impact of the tenure promotion process on the perception of public engagement, (2) faculty using extension as a comparative tool for understanding levels of engagement, (3) the perception of a changing culture of public engagement motivated by graduate students and junior faculty, and (4) the role that mentorship plays in encouraging or discouraging participation in public engagement activities. We did not find any meaningful differences between pre-tenure and tenured faculty, or between faculty from high engagement and low engagement departments. This may be simply because the sample size was too small to permit inter-group comparisons in the face of predictable variability in response and experience. Thus, findings are presented for the focus groups in the aggregate, unless otherwise noted, and with quotes including group identification (i.e., PT or T for tenure status and high or low for engagement level).

#### Motivations are mostly personal, with some factors relating to the culture of public engagement

When we asked participants, “what motivates you to connect with the public,” the responses mostly reflected intrinsic motivations. Several participants expressed a feeling of personal responsibility, obligation, or sense of duty to engage with the public. In some cases, participants specified that this sense of responsibility stemmed from their position as a faculty member at a *public* institution:

I think that as a public institution, supported by state taxpayers, we have an obligation to engage the public, to engage the constituents of this state. And I think it’s part and parcel of being a professor at a public university. So that’s one thing that motivates me. (T-high)

Similarly, several participants described public engagement as being an ethical or moral imperative. In this case, the experiences mentioned were less a result of their position within a public institution, but rather a personal sense of duty to share their expertise and knowledge. One participant described this motivation as being tied to their role as a citizen, describing their participation in public engagement as “morally important” because of their role as an expert (PT-high). These findings align with previous research on motivations to engage based on the idea that engagement, in and of itself, is a public good [25]. Another motivation briefly noted was a sense of personal satisfaction, with public engagement described as “fun” or in terms of “enjoyment.” Although only two participants noted this motivation, a sense of personal satisfaction has also been explored in previous research as a motivating factor [24].

While most motivations discussed were personal, there were references to extrinsic motivations, specifically related to the culture of public engagement. Additionally, one participant described grant funding requirements for outreach as a motivation, but more often than not, funding was referenced as a barrier, which we discuss further below. As for culture, participants often described the culture of public engagement as inherent to their university and department, recognizing a sense of commitment from their colleagues to engaging with the community. Participants acknowledged their colleagues’ participation in engagement activities as a motivating factor in addition to the university’s land-grant status:

But good grief, the college bends over backwards to help people to engage the public in terms of their research and scholarship. We are not only a public university, we are a land-grant university. That makes all the difference in the world. (T-high)

The influence that the culture of public engagement has on willingness to engage was also framed as something that needs ongoing work and support at the institutional level. In these cases, fostering a supportive culture around public engagement at the university and within departments was described as needing a clearer framework and specified resources. References to the culture of public engagement appear throughout these discussions, often along with institutional factors, which we discuss in the following sections.

#### Barriers center around time, resources, and concerns for reputation

To understand barriers that impact willingness to engage with the public, we specifically prompted discussion about both institutional and non-institutional barriers. Our findings largely replicate obstacles identified elsewhere in the literature. The institutional factors that were considered barriers were often brought up in the context of tenure requirements, with some perceptions of lacking institutional structure and support for sustained opportunities to engage. A few other barriers were commonly noted including a perception of limited time and resources, concern for backlash when engaging on social media, and concern for managing public perceptions of academic expertise.

Concerns about time and resources are often examined in public engagement research, including the perception that engaging with the public is an opportunity cost that takes away from research or teaching responsibilities [10]. In line with this work, these discussions highlighted concerns about being able to respond quickly enough when asked to engage and feeling like there is no time to engage because it is not considered as valuable as other expectations. For example, one participant noted, “…when the greatest value is measured on things like publications and your teaching, finding the time to also fit in meaningful public outreach is challenging” (PT-low).

When prompted about engagement on social media platforms, like Twitter or Facebook, some participants expressed concern about finding a balance between promoting their work and expressing their personal views. There were mentions of the fear of being labeled as ideological or posting something that could result in backlash from within the university:

I would argue, at least right now, that I never feel like I’m engaging as a private citizen, that I’m always representing the university. And so I’m very careful about what I choose to post, whether it’s Twitter. I’m not on Facebook. But I think about it in terms of retribution, in terms of tenure reviews. (PT-low)

Participants also discussed a sense of tension between sharing their knowledge with the public and the fear of “skepticism from the community” (PT-low). The concern for backlash and skepticism suggests an awareness of how public engagement can have potentially negative impacts on faculty reputation and credibility. This can create a sense of pressure around crafting their message carefully in order to be perceived as credible:

So some people say, “Oh, you’re faculty at [*university name*], you know, you speak the truth.” And other people are very skeptical. And especially in my line of work, I have internalized that it’s very important to me that I be perceived as credible by a wide swath of people. (PT-high)

#### New goals of engagement referenced without prompting

As mentioned earlier, in addition to the broad consideration of factors that influence willingness to engage, new research provides a goal-based framework outlining seven goals of public engagement with science [4]. These include avoiding potential controversy, educating the public, building democratic capacity through deliberation, widening the representation of voices, soliciting input on value debates triggered by science, enabling responsible innovation, and shaping policy. While we did not prompt discussion about these goals specifically, they were all were mentioned at least once (RQ4). Both pre-tenure and tenured faculty provided examples that aligned with several of the goals, most often when prompted about their perceptions of what communicating with the public looks like and which audiences they were most interested to engage with. The goals that most frequently appeared were *shaping policy* and *educating the public*, as well as several examples broadly related to the goals of *enabling responsible innovation* and *widening the representation of voices*.

Engagement related to *policy shaping*, included a desire to reach “decision-makers.” Participants expressed how engagement with decision-makers was a way to influence their understanding of and influence over how issues related to the participants’ research are governed. This goal was often associated with a desire to make an impact, recognizing the value of engaging with decision-makers that influence policy initiatives:

Because actually, like thinking back, like my original motivation for even going to grad school was basically to help support and provide research that will inform better policy. So, I mean, like from my perspective, something that will have like broader systemic change would be like the ultimate communication to the extent that that’s possible. (PT-high)

Participants also cited several examples of engagement activities related to the policy-shaping goal that they either had participated in or wanted to. These forms of policy-oriented engagement included contributing to amicus curiae briefs to the state Supreme Court, commenting on federal rules for regulatory departments, talking with local policy representatives about county-level issues, or providing testimony and participating in committee meetings when invited as an expert.

In addition to shaping policy, participants regularly cited the importance of *educating the public* as a goal of public engagement. Many examples were directly focused on ensuring different publics had information about specific issues, such as details about COVID-19 and about “sharing the knowledge” that they have as experts. Participants also described educating the public about the value and nature of science more broadly, with an interest in building excitement and appreciation for science:

I think the purposes of public engagement must include, strongly include, achieving a level of science literacy. And by science literacy, I don’t mean developing expertise in science, but developing a sense of appreciation of science. (T-high)

I’m always willing to share my expertise … but the more important message … is communicating the nature of science and how it works, because there’s a real lack of understanding of that pretty broadly. (T-high)

Some of these perspectives represent a knowledge-deficit model of thinking. For example, engagement to increase science literacy or change policy in line with what researchers think is best for the public as the ultimate goal of communication represents a knowledge-deficit model of thinking. This runs counter to the idea that collaboration and engagement with various publics can facilitate a connection between science and society.

In contrast to more knowledge-deficit modes of engagement, participants also discussed experiences related to *enabling responsible innovation* and *widening the representation of voices*. The distinction between these throughout the discussions was not always clear, but participants noted the importance of considering the perspectives of affected publics and, in some cases, collaborating closely with various stakeholders in their research. Some participants discussed various publics that they had engaged with, expressing how in these interactions they were “not experts anymore” but instead were “learning from them” (T-low). Other participants provided specific examples of working with affected publics regarding research on treatments for diseases (PT-low) or collaborating with industry stakeholders (T-low). While these goals defined by previous research were found, there were no instances of other goals, not included in previous research, emerging from these conversations (RQ5).

#### Theme 1: Expectations surrounding tenure promotion can be restricting

While these examples lend additional context and validity to previous research on public engagement, a primary aim of conducting these focus groups was to find themes relevant to the current state of faculty experiences with public engagement.

The first prominent theme that emerged from these discussions was the sentiment that the tenure promotion process can be a barrier to engaging with the public. Tenure and promotion at U.S. public universities are often based on excellence in three main categories: research, teaching, and service [26]. Participating in public engagement activities falls into the service category, along with university-focused service such as sitting on university committees. What the discussions uncovered was a perception that, while public engagement may be valued within a department, it is not considered as a serious component of the expectations that faculty face professionally. This was highlighted by participants’ experiences with tenure and the expressed understanding that what matters for tenure and promotion is research and teaching, whereas public engagement activities were “icing on the cake” (PT-high) or generally just “not a determining factor in promotion decisions” (T-low):

I know what a publication does to my tenure file. I don’t know what public engagement does to my tenure file. And so that’s unclear to me. (PT-low)

The question, of course, as everyone here has pointed out is how much of it I should do as an assistant professor and how much of it is traditionally counted within what would count towards tenure. (PT-low)

Beyond the lack of clarity about how public engagement is valued in tenure and promotion decisions, some participants expressed the idea that public engagement is a secondary activity that they choose to do if they have the opportunity and time:

…for me, it’s more about like deciding which requests to respond to when I also have to, you know, meet tenure obligations. (PT-high)

You know, again, something that we’re sharing here is that, you know, it’s kind of this idea that it’s a kind of unacknowledged, or expected part of what we do, that we’re not directly acknowledged for, you know, and paid for. (PT-high)

Participants further expressed a sense of discouragement to engage, specifically as a pre-tenure faculty member. For example, one participant claimed that “there’s an inherent discouraging pressure on reaching out to the public” because when dossiers are evaluated, participation in university committees are considered “enough” for service and so the expectation is to “spend more time in your lab, spend more time on your research, work on developing your teaching” (T-high). In this sense, participants explained how tenure guidelines and expectations inhibit and discourage public engagement by junior faculty, thereby effectively reserving it as something only tenured faculty can do:

I would say that tenure is essential for allowing researchers to get public engagement, because it takes more time. You can’t do research as quickly if you’re first asking the public about it. (PT-low)

The perception of tenure as a barrier did not resonate with all participating faculty and was not necessarily specific to the university, but as an issue in academia more broadly. One participant exclaimed that they “don’t necessarily feel a very huge tension or pressure not to engage” noting that their department was “quite supportive” (PT-low). However, others emphasized a culture in which engagement does not seem adequately valued. In conversations with both tenured and pre-tenure faculty, participants discussed a need for a cultural shift in which engagement is valued and supported by the institution. One participant noted that not everybody needs to engage, but “some people need to be not only allowed to do it but encouraged to do it” (T-low). The perception expressed here is that those who want to engage and are good at it should be supported and acknowledged for that work.

#### Theme 2: Extension as a point of comparison

Another prominent theme in the discussions was a reference to the engagement practices of faculty members with extension appointments as a point of comparison. As previously noted, extension appointments at land-grant universities require some form of engagement with communities throughout the state [41]. Discussions that mentioned extension referred to it as a point of reference for participants’ understanding of their engagement and regarding the clarity of expectations around engagement. These conversations were only found in three of the four focus groups. Extension was *not* mentioned in the pre-tenure low-engagement group.

When participants noted extension, they often alluded to a sentiment that although they might not engage with the public *like extension faculty*, they still do something:

Many professors have extension appointments, which, … they have this very clear idea of outreach as professional vocation in extension work. So I don’t have an extension position, but even in the last two weeks or so, you know, I did a TV interview, and I did a live radio interview, because I was the person who answered the phone, and that’s sort of the culture in the field. (PT-high)

Participants also expressed some uncertainty about expectations around public engagement and suggested that they did not perceive public engagement as their responsibility because of the existence of extension faculty. What seemed confusing for participants was the lack of the clearly defined role that engagement plays in their jobs, as compared to extension faculty where those expectations are defined, understood, and put into practice. This is related to the perception that public engagement work is not their job, but rather specifically the job of extension faculty. However, the boundaries of this distinction are blurred by the fact that some faculty do choose to participate in engagement, but that engagement is “more part of the ethos” rather than “baked into the system” of their roles (T-high):

I don’t have this clear well-defined message that I feel needs to get out there, that I need to push about my research or its relevance. I have things that I think I might be able to add, but, yeah, especially comparing and contrasting with people who have extension programs, that’s not what my job is about. (PT-high)

For example, I don’t have an extension component in my appointment, so I am in research and teaching. But we do have individuals that are exactly focused on public engagement and extension and talking to public, but in different levels also. (T-low)

#### Theme 3: Graduate students and pre-tenure faculty are driving a changing culture of public engagement

The third prominent theme in these discussions focused on how graduate students and junior faculty who are interested in public engagement are embracing the changing culture surrounding it, deviating from the perceived status quo of university culture. The interest in public engagement by graduate students and junior faculty is described in terms of personal interest and departmental change. This sentiment included examples of experiences that indicate how junior faculty and graduate students are seeking out and excited about engagement opportunities:

I think the role of the students cannot be neglected. I have had specifically students who told me, “In my Ph.D., I want to engage in outreach activities.” And we have been discussing this point. So I really think that one direction of questions to ask would be how students and the students’ interests play into this question. (PT-high)

And I know that for some of my younger colleagues, it is also a factor for some of them, that motivates them, to do public outreach. And for some of the recent graduates of our program, at least in my subspecialty, they’re motivated by a desire to listen to marginalized populations and to do research that will be meaningful and that will actively engage those marginalized populations. (T-high)

The interest in engagement was also presented as a pressure that these groups put on departments and senior faculty. However, in one particular case expectations about engagement opportunities and resources received push back from senior faculty, as recounted by one of the participant’s experiences:

I remember at one point, a grad student had brought in some requests from the graduate body to my department, and they suggested more public engagement on the part of faculty. And my colleagues were really upset because they were very threatened by this. It seemed to be somebody telling them how to do their scholarship. And I actually think we shouldn’t overestimate, we shouldn’t underestimate, rather, the level of hostility that we can incur from senior faculty who don’t like being guided in new directions. (T-low)

Throughout these discussions emerged a critique of the culture surrounding engagement in which incentives are lacking, directly inhibiting wider participation. When institutional culture was brought up in these conversations, participants posed questions and provided suggestions about ways in which engagement could be more systematically included to meet the needs of junior faculty and graduate students. These included ideas about expectations of engagement “as part of the training process of grad students” (PT-high) as well as suggestions that there be a school or university level “mandate” on how to “count and evaluate this type of participation” (T-low). Furthermore, participants made the case for why incentivizing junior faculty and graduate students would be a positive thing, for them and for universities more broadly:

We could actually really better reflect the diversity of the community we serve if we were better at incentivizing younger, pre-tenured, or even graduate student scholars to be the kind of face of the university. (T-low)

I agree that our younger people, who are far more diverse, and far more able to go with multimedia platforms, are really powerful and effective in their smaller circles, but they don’t get the opportunities to really come up to a higher audience. (T- low)

#### Theme 4: Mentorship matters for encouraging or discouraging public engagement

The last notable theme from these discussions is the influence that mentorship has on faculty members’ decisions about whether to engage. There were examples of experiences in which mentors were seen as both encouraging and discouraging of participation in various engagement activities. Aligned with the perceptions of motivations described earlier, participants highlighted how their colleagues and mentors can act as role models to encourage engagement:

When you see colleagues out doing incredible engagement, out doing incredible research, and it’s normal and expected, it’s, to me, very motivating. It’s something that you want to be a part of so that you can keep up with your colleagues, and outside of the responsibility of doing it, it’s just sort of what’s done. So you take that on because that’s the role that’s sort of provided for you. (PT-high)

Participants more often, however, expressed a sentiment that participating in public engagement is actively discouraged–relating personal experiences to previously discussed barriers like tenure and promotion. Some participants noted a tendency they have observed or personally engaged in, to provide a sort of caution to their students or mentees. This caution stresses the importance of what matters for promotion and highlighting that choosing to focus on engagement can have consequences for their professional careers:

I do see it with my colleagues mentoring younger faculty in the department, and strongly discouraging them from doing anything but their narrow area of research. (T-high)

This tendency suggests that the status quo of how engagement is valued is persistent, although there are perceptions that it is changing. For example, while one participant noted that, as a mentor for a new faculty member, they recognized that the “attitude [of engagement] is transforming,” they also noted that many of the “older faculty” that are currently in mentorship roles are not aware of this transformation and instead “go by the old guidelines” (T-high). This dynamic between what new and emerging academic faculty want and value in their professional careers and that of many established, tenured faculty indicates a tension emerging from a potential broader cultural change in academia regarding public engagement.

## Discussion

The findings from these four focus group discussions provide additional context about individual faculty perspectives and lived experiences with public engagement, specifically related to factors that influence faculty members’ willingness to engage. These discussions also highlighted emerging themes that further contextualize the state of public engagement and what next steps are necessary to encourage broader engagement. Each of the main themes touches on aspects of a changing culture surrounding public engagement as well as the institutional factors that influence perceptions of and willingness to participate in engagement activities. Despite these findings being exploratory in nature, they signal ongoing shifts that deserve attention with additional focused research.

Before turning to the details of our findings, it is important to understand the nature of the data presented here. Our in-depth focus groups represent a very small set of perspectives from a single land-grant university. Therefore, these findings are not causal or generalizable across all land-grant university faculty. Instead, the perspectives and experiences exposed in these discussions provide a more nuanced and rich exploration of what may be changing or unknown that should be further examined. Additionally, because the transcripts were anonymized before we analyzed them, it was unclear which participants were speaking at any given time. Therefore, it is possible that participants who spoke more frequently or repeated viewpoints in different ways may be overly represented in some of our findings. However, the focus groups were conducted by a professional moderator who managed the discussion to allocate equal speaking time. Additionally, since the aim of this qualitative approach is based on contextualizing current understandings of public engagement in scholarship with faculty experiences and perspectives, these limitations do not reduce the value of our findings.

### Graduate students and pre-tenure faculty as drivers towards transformation

The themes that center around mentorship and the interest in public engagement by graduate students and pre-tenure faculty suggest that there may be a shift in the culture surrounding public engagement in higher education. At the same time, our findings about perceptions of tenure promotion and the role of extension provide insight into the prominence of the cultural status quo. As interest in public engagement from the broader scientific community continues to grow, cultural change at the university level may have a lasting impact on whether scientists choose to participate in engagement activities. The potential influence of graduate students and pre-tenure faculty on the culture of engagement in some departments should be further explored. Previous research on behavioral and cultural socialization may provide guidance for understanding what forces might drive a cultural change in higher education institutions.

Research on political socialization considers how individuals’ behaviors can be influenced by exposure to socialization efforts that specifically target political participation, like voting [See 46–49]. Much research in this space explores how socializing children can influence the attitudes and behaviors of adults. For example, one study examined the effect of the Kansas kids voting program, a program aimed at educating children about voting. They found higher voter turnout rates among their parents of children participating in this program [50]. This example of “trickle up” socialization emphasizes what is identified in cultural socialization between children and their parents in how a child can positively influence an adult to make meaningful behavioral changes [51]. This relationship has also been examined in the context of engagement with progressive cultural issues, such as acceptance of transgender rights [52]. Furthermore, research in the field of higher education explores socialization with regard to socializing faculty and graduate students into department and university cultures [14, 53–55]. In a study of early career scholars, graduate work and mentorship influenced individuals’ interest in publicly engaged scholarship as faculty members [56]. These findings could relate to the motivations of graduate students and junior faculty that may be having a sort of “trickle up” effect on tenured faculty in their department, and perhaps administrators or other university-level decision makers. Additional research in this space should expand upon how graduate students and post-doctoral researchers perceive public engagement, as well as how pre-tenure faculty are engaging differently from their tenured colleagues.

### Institutional factors as gatekeepers to the value of engagement

Another topic that received substantial attention in the focus group discussions centered around tenure and promotion and expectations of faculty being discouraged from participation in public engagement activities. These perspectives and experiences were especially evident when participants discussed barriers to engagement, extension, and mentorship. While not every faculty member in these discussions felt that they were discouraged or disincentivized from participating in public engagement, many noted the pressure they felt to focus on research and teaching, rather than public engagement for the sake of tenure and promotion requirements. Faculty also expressed how engagement was not practical or even possible in some cases until one is tenured. Personal experiences shared in these discussions revealed that, in many cases, these pressures resulted in active discouragement by mentors of graduate students and junior faculty. The discussions about extension also showed that some faculty members do not consider engagement to be part of their job unless there are specific expectations for it built into their job description.

This theme of institutional culture creating barriers to engagement is not new. In fact, several studies, especially in higher education research, have discussed institutional culture as presenting barriers to engagement [See, e.g., 57–62]. Scholars in the public engagement with science space have similarly expressed the need for greater institutional support for engagement activities [e.g., 9, 11, 15, 16]. However, the perception by faculty that public engagement is not valued for tenure and promotion, and therefore discouraged by mentors, is still relevant. This underscores the urgency for practical insight into how institutions can play an active role in finding ways to incentivize (rather than disincentivize) participation in engagement activities. The current culture of academe prioritizes research over public engagement and recent arguments have been made that this culture must change in a way that values the engaged scholar and prioritizes research that can reach a broader audience and inform public debates [63].

Universities play an important role in shaping the culture of public engagement across departments. The institution of higher education more broadly can also influence cultural change around engagement by encouraging specific ways that universities can value and incentivize participation in engagement. Three general approaches that might be considered at the institutional level to support public engagement include a top-down approach, investment from the top in supporting a bottom-up approach, or direct investment in engagement at the “bottom.” These approaches can be considered to encourage universities to recognize the shifting culture around public engagement and support the shift institutionally.

A top-down approach might include changes to tenure and promotion guidelines, the allocation of funding and guidance for departments to support public engagement, or even coalitions across universities to promote public engagement. Furthermore, faculty contracts could specify the role of public engagement in service requirements.

Bottom-up investment could include supporting inclusive engagement opportunities that connect academic faculty with various publics in collaborations that meaningfully contribute to scientific knowledge and inquiry. A model example is the Civic Science Fellows program in which postdoctoral fellows from diverse backgrounds collaborate with partners and organizations in various fields “with the aim of creating effective new forms of collaboration, outreach, and communication to strengthen widespread engagement with science and its relationship to broader society” [64]. This program takes an innovative approach to addressing a broad range of challenges that continue to emerge at the intersection of science and society.

Even more direct approaches might invest in the “bottom,” which could include creating PhD minors in science communication and promoting their value to graduate students across science fields. Other direct investments might create and support science communication groups on campus, requiring or incentivizing science communication training, or including service expectations in graduate programs. Several universities already have the infrastructure for these investments in incentivizing engagement. For example, in addition to supporting student-run science communication groups and hosting engagement events, the University of Wisconsin-Madison provides a PhD minor as well as graduate and undergraduate certificate programs through the Department of Life Sciences Communication that expose students to engagement early on in their academic and professional science careers [65]. Similarly, Arizona State University provides the option of a graduate certificate in science communication [66]. To pique the interest of emerging scholars at the undergraduate level, Cornell University offers an undergraduate science communication minor [67].

Regardless of what approaches universities take to support public engagement with science, the effectiveness of these approaches in encouraging faculty to participate requires further research to meet the calls from the scientific community. Additionally, our findings suggest that there is a need to reiterate the range of forms of engagement and further address a tendency for some faculty to view engagement from a knowledge-deficit perspective. Focus groups alone do not reflect the full range of engagement perspectives that are represented in the literature [e.g., 18]. Incentivizing engagement practices towards genuine engagement *with* various publics requires that the work of engagement scholars is accessible in a way that encourages systematic change and highlights the importance of a range of engagement practices. This could mend the forming disconnect between the theory that thoroughly explains models of engagement and what is conducted in practice. Such efforts have the potential to avoid the situation in which faculty intentions to engage meaningfully result in reiterations of ineffective deficit model engagement [e.g., 68].

The focus groups we conducted highlight that there is a desire among faculty to engage meaningfully with the public, but that there are also genuine barriers that extend beyond individual faculty decisions to participate. These focus groups further underscore the value of qualitative approaches in public engagement research. By exposing examples of the experiences of faculty from their point of view, this study contributes to a more well-rounded understanding of the context surrounding the broader needs in public engagement, from which we can develop strategies to encourage and support interested faculty in meaningful public engagement.

## Supporting information

S1 Appendix Focus group facilitation script and questionnaire. (DOCX)
