# Supplementary material for: Public engagement: Faculty lived experiences and perspectives underscore barriers and a changing culture in academia
Source: PLoS One. 2022 Jun 15;17(6):e0269949. doi: 10.1371/journal.pone.0269949 (PMC9200360; doi:10.1371/journal.pone.0269949)
Supplement: S1 Appendix — (DOCX) [file pone.0269949.s001.docx]

Supplementary Information

**Public engagement: Faculty lived experiences and perspectives underscore barriers and a changing culture in academia**

This document includes:

S1 Appendix: Focus group facilitation script and questionnaire

**S1 Appendix: Focus group facilitation script and questionnaire**

INTRO

**(6 minutes)**

Hello and welcome. My name is Ken Croes. I’m a research project director here at the UW Survey Center and I’ll be moderating our group today.

We’re joined by _______ from _____, who is a student and will be observing.

Thank you all for being here. This group is being held as part of a NSF-funded research collaboration between the Wisconsin Institute for Discovery, WARF, and the Department of Life Sciences Communications.

We’ve invited you to be part of this group in your capacity as UW–Madison tenured/non-tenured faculty member. The purpose of the group is to learn how faculty regard public outreach, engagement, the Wisconsin Idea, etc. We’ve invited you to be here whether or not you engage in such activities. We expect we’ll have a wide variety of experiences and viewpoints here today, and all points of view are important to this research.

I have a few notes to get us oriented:

- I’m audio recording so that we don’t miss any of your comments. No names will be used in any reports. Your comments are confidential. I ask that you too maintain confidentiality by not repeating what is said here after the group.
- Your participation is voluntary. If there’s any question you'd prefer not to answer, that’s fine.
- I ask that only one person talk at a time. But please feel free to agree, disagree, follow-up on what others have said.
- My role is to introduce questions and make sure everyone has a chance to talk. To cover all the questions, there may be times when I need to move us to the next topic. I ask for your indulgence in advance if that happens.

With all that in mind, let’s go around the table for introductions. Please say your name, department, and what motivated you to be here today.

1. What is engagement

OBJECTIVES AND/OR BACKGROUND:

- We want to understand how participants define public engagement and what activities they regard as public engagement activities – what types of events, their experiences, what audiences they’ve interacted with, how engagement activities differ, etc.

**(5 minutes)**

You may have heard discussions about engagement, outreach, the Wisconsin Idea, science communication, public scholarship, etc. -- these are some of the ways academics talk about connecting with different communities and audiences.

We’d like to begin by getting a sense of what you see as **engagement with the public**. What comes to mind when you think of scholars connecting or communicating with the public?

2. Factors that impact someone's willingness to engage

OBJECTIVES AND/OR BACKGROUND:

- We want to get at what are motivations and barriers to participate. We anticipate that high and low engagement groups will respond to these questions differently.
- In addition, we are interested in the participants’ perceived outcomes of public engagement activities

(40 minutes)

Next, we’re interested in the reasons why academics pursue public engagement or not.

What motivates you to connect with the public?

For those of you who do not pursue public engagement, can you say why you do not pursue it?

How does the culture of your department, college, or at UW more broadly affect public engagement?

Thinking beyond institutional barriers here at UW, what other challenges have you faced when trying to engage with the public?

Does the tenure system at the UW-Madison have incentives or disincentives for public engagement?

IF YES: What are they?

Do you think the UW differs from other universities in terms of the incentives or disincentives for public engagement that are built in to the tenure system?

IF DIFFERS, PROBE: How does UW differ?

3. Audience

OBJECTIVES AND/OR BACKGROUND:

- We are interested in learning about which audiences they think of when they think of public engagement and which they are interested in connecting with.

(25 minutes)

What groups, audiences, or communities do you think of when you think of public engagement?

Are you interested in connecting with certain groups or audiences over others? IF YES: Which ones and why?

Some people discuss engagement as going in one direction – from academics and scholars to the public. How does the public affect academic engagement?

Are there other ways your work and scholarship are informed or affected by public engagement?

IF YES, PROBE: What are they?

**WRAP-UP QUESTION**

(4 minutes)

Is there anything we should’ve asked you that we haven’t regarding academic engagement with the public – any additional closing comments you’d like to make?
